# Toward Highly Selective Electrochemical CO_2_ Reduction using Metal‐Free Heteroatom‐Doped Carbon

**DOI:** 10.1002/advs.202001002

**Published:** 2020-06-30

**Authors:** Binbin Pan, Xiaorong Zhu, Yunling Wu, Tongchao Liu, Xuanxuan Bi, Kun Feng, Na Han, Jun Zhong, Jun Lu, Yafei Li, Yanguang Li

**Affiliations:** ^1^ Institute of Functional Nano & Soft Materials (FUNSOM) Jiangsu Key Laboratory for Carbon‐Based Functional Materials and Devices Soochow University Suzhou 215123 China; ^2^ College of Chemistry and Materials Science Nanjing Normal University Nanjing 210023 China; ^3^ Chemical Sciences and Engineering Division Argonne National Laboratory Lemont IL 60439 USA; ^4^ School of Advanced Materials Shenzhen Graduate School Peking University Shenzhen 518055 China

**Keywords:** CO_2_ reduction, heteroatom codoping, mesoporous carbon, selectivity

## Abstract

There are growing interests in metal‐free heteroatom‐doped carbons for electrochemical CO_2_ reduction. Previous studies extensively focus on the effect of N‐doping, and their products severely suffer from low current density (mostly <2 mA cm^−2^) and limited selectivity (<90%). Here, it is reported that heteroatom codoping offers a promising solution to the above challenge. As a proof of concept, N,P‐codoped mesoporous carbon is prepared by annealing phytic‐acid‐functionalized ZIF‐8 in NH_3_. In CO_2_‐saturated 0.5 m NaHCO_3_, the catalyst enables CO_2_ reduction to CO with great selectivity close to 100% and large CO partial current density (≈8 mA cm^−2^), which are, to the best of knowledge, superior to all other relevant competitors. Theoretical simulations show that the improved activity and selectivity are stemmed from the enhanced surface adsorption of *COOH and *CO intermediates as a result of the synergy of N and P codoping.

## Graphical abstract

High surface area N,P‐codoped mesoporous carbon is prepared from the NH_3_ annealing of phytic acid functionalized ZIF‐8. It can catalyze the CO_2_ reduction to CO with great selectivity close to unity and large CO partial current density, superior to all existing metal‐free heteroatom‐doped carbons to the best of knowledge.

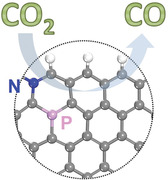

Electrochemical CO_2_ reduction has the potential to transform atmospheric CO_2_ to value‐added chemical fuels or industrial feedstocks, and represents a promising means to close the natural carbon cycle.^[^ ^1^, ^2^ ^]^ Its main challenge lies in the development of efficient electrocatalysts with simultaneously high activity, selectivity and stability, and preferably made of earth‐abundant materials.^[^ ^3^, ^4^, ^5^, ^6^, ^7^ ^]^ Among many different candidates, metal‐free carbonaceous materials are the most appealing due to their relatively low costs.^[^ ^8^, ^9^, ^10^ ^]^ Even though pristine carbon is usually electrochemically inert, heteroatom doping can significantly modify its local electronic configurations or introduce structural defects, and thereby afford it with improved electrocatalytic activities.^[^ ^11^ ^]^ Over the past decade, heteroatom (e.g., N, B, P, and S) doped carbons have been widely investigated for important electrochemical reactions including such as hydrogen evolution reaction (HER), oxygen reduction reaction (ORR) and oxygen evolution reaction (OER).^[^ ^12^, ^13^, ^14^ ^]^ Their application in CO_2_ reduction reaction (CO_2_RR) was first reported by Salehi‐Khojin and coworkers in 2013 and since then has attracted growing interest.^[^ ^15^ ^]^ Most current research efforts are focused on N‐doped carbons with CO as the main reduction product,^[^ ^16^, ^17^, ^18^, ^19^, ^20^, ^21^ ^]^ even though there are also sporadic reports about other singly doped (e.g., F or P‐doped) carbons.^[^ ^22^, ^23^ ^]^ Unfortunately, all these heteroatom‐doped carbons suffer from limited activity and selectivity. Their peak CO Faradic efficiency is less than 90% and their CO partial current density is less than 5 mA cm^−2^ (most commonly <2 mA cm^−2^) even at very negative potentials.^[^ ^8^, ^9^, ^10^ ^]^ Theoretical simulations indicate that graphitic N, pyridinic N or pyrrolic N sites in N‐doped carbons all bind *COOH (the intermediate to CO) too weakly.^[^ ^16^, ^17^, ^18^ ^]^ To further improve the activity and selectivity would require strategies to better tune their electronic structure and enhance the *COOH adsorption.

We reason that one possible approach is to pursue the codoping of two different heteroatoms. The introduction of a second dopant in addition to N may add another dimension of tunability over the local electronic structure. Unexpected electrocatalytic activity may result from the synergistic coupling between two dopants. The effect of codoping in carbonaceous materials has been explored for ORR, OER, and HER,^[^ ^12^, ^24^ ^]^ but is far less studied for CO_2_RR. One of the very few examples is from Lin and coworkers showing that N,S‐codoped carbon nanofibers had an improved CO_2_RR performance.^[^ ^25^ ^]^ However, its peak CO faradaic efficiency of 94% was not achieved until at ≈0.7 V versus reversible hydrogen electrode (RHE), corresponding to large overpotential of *η* ∼ 600 mV.

Herein, we report that N,P‐codoping dramatically boosts the CO_2_RR performance. As a proof of concept, high surface area N,P‐codoped mesoporous carbon is prepared from the high‐temperature NH_3_ annealing of phytic‐acid‐functionalized ZIF‐8 metal organic frameworks (MOFs). The resultant product exhibits selectivity close to unity at low overpotential and large CO partial current density. Such excellent activity and selectivity are understood via theoretical calculations showing that the synergy between N and P significantly stabilizes *COOH and *CO intermediates.

**Figure** **1**a schematically illustrates our three‐step synthetic procedure toward N,P‐codoped mesoporous carbon (see the Supporting Information for details). ZIF‐8 was first prepared from the self‐assembly of Zn^2+^ ions and 2‐methylimidazole in methanol. The formation of crystalline MOF structure is confirmed by its X‐ray diffraction (XRD) pattern in agreement with the simulated result (Figure S1, Supporting Information). Scanning electron microscopy (SEM) imaging reveals that ZIF‐8 crystals have a dodecahedral shape with smooth outer surface and an average size of ≈1 µm (Figure 1b). For the second step, ZIF‐8 was functionalized with different amounts of phytic acid by soaking in its aqueous solution (Table S1, Supporting Information). Phytic acid is selected as the phosphorus precursor here since it contains abundant phosphate groups, and can selectively adsorb on the ZIF‐8 surface presumably via their interactions with the N‐containing moieties of ZIF‐8. The functionalization causes no obvious morphology change (Figure S2, Supporting Information). Subsequently, phytic‐acid‐functionalized ZIF‐8 was annealed at 1050 °C under the NH_3_ atmosphere. Zn species evaporates under such a high temperature,^[^ ^26^ ^]^ leaving behind N,P‐codoped mesoporous carbon (denoted as N,P‐mC). For control experiments, singly N‐doped mesoporous carbon (denoted as N‐mC) was prepared without the phytic acid functionalization under otherwise identical conditions. All products are analyzed to be amorphous by XRD (Figure S1, Supporting Information). N,P‐mC (prepared with the standard amount of phytic acid, the same hereinafter unless otherwise specified) is observed to have the concave dodecahedral shape with a shrunk size of ≈600 nm under SEM (Figure 1c,d). Interestingly, we find that the unique concavity is afforded by the phytic acid functionalization since N‐mC only consists of carbonaceous dodecahedra (Figure S3, Supporting Information). When the added amount of phytic acid is gradually increased, the final product becomes increasingly concave. However, adding too much phytic acid causes these concave dodecahedra to be partly damaged (Figure S3, Supporting Information). Transmission electron microscopy (TEM) examination of N,P‐mC corroborates their amorphous nature and the existence of abundant mesopores (Figure 1e,f). Energy dispersive spectroscopy (EDS) elemental mapping of C, N, and P supports their uniform spatial distribution in N,P‐mC (Figure 1g–j). Moreover, N_2_ adsorption‐desorption measurements show that N,P‐mC and N‐mC have significant surface areas of 2325 and 2871 m^2^ g^−1^ as well as large pore volumes of 1.25 and 1.51 cm^3^ g^−1^, respectively (Figure S4, Supporting Information). Their pore size is analyzed to distribute between 2–5 nm. Such advantageous structural features are presumably afforded by the Zn evaporation and the NH_3_ etching of the carbonaceous skeleton. They are beneficial to electrochemical applications.

**Figure 1:**
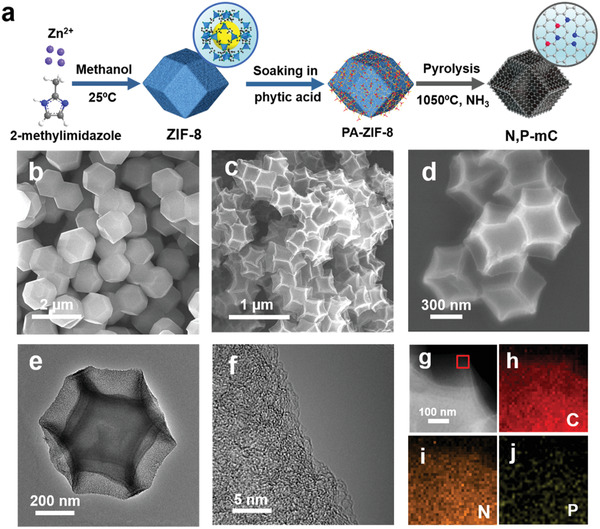
Preparation and microscopic characterizations of N,P‐mC. a) Schematic synthetic procedure toward N,P‐mC; b) SEM image of ZIF‐8; c,d) SEM images and e,f) TEM images of N,P‐mC at different magnifications; g) STEM image and h–j) EDS elemental mapping of the enclosed area in (g) for C, N, P in N,P‐mC.

N,P‐mC is further interrogated with multiple spectroscopic characterizations to probe its bonding configurations. The Raman spectrum exhibits pronounced D and G bands, evidencing that it is partially graphitic (Figure S5, Supporting Information). Deconvolution of the C 1s X‐ray photoelectron spectroscopy (XPS) spectrum unveils the contribution of C–N/C–P bonds at 287.4 eV (**Figure** **2**a).^[^ ^27^ ^]^ Rich pyridinic, pyrrolic, graphitic, and oxidized N functionalities are detected from N 1s XPS spectrum (Figure 2b).^[^ ^28^ ^]^ Direct C–P bonding is reflected from the main peak at 132.5 eV of the P 2p XPS spectrum (Figure 2c).^[^ ^29^ ^]^ Quantitative analysis unveils that the N and P contents in N,P‐mC are 5.3 and 0.11 at%, respectively. The low P at% is due to the high annealing temperature (1050 °C) but its concentration is reproducible. Similarly low levels of P doping were previously reported in literatures.^[^ ^29^, ^30^, ^31^ ^]^ When different amounts of phytic acid are added, the P doping level in the final products can be tuned between 0.04 and 0.24 at%, while their N at% generally decreases with the increasing P at% (Figure S6 and Table S2, Supporting Information). Moreover, consistent results are garnered from X‐ray absorption near edge structure (XANES) analysis. The C K‐edge XANES of N,P‐mC exhibits a weak but discernible peak at 287.8 eV assignable to carbon‐heteroatom (e.g., C–N or C–P) bonds (Figure 2d).^[^ ^24^ ^]^ Its N K‐edge XANES features the main signals from pyridinic and graphitic N (Figure 2e).^[^ ^24^ ^]^ The formation of C–P bonds is again confirmed from the P L_2,3_‐edge XANES showing the π* signal at 132.2 eV (Figure 2f).^[^ ^32^, ^33^ ^]^ Detailed spectroscopic characterizations were likewise carried out on N‐mC, and the results are summarized in Figure S7 in the Supporting Information. Not surprisingly, N‐mC is shown to be free of P. Its C and N XPS and XANES spectra are similar to those of N,P‐mC.

**Figure 2:**
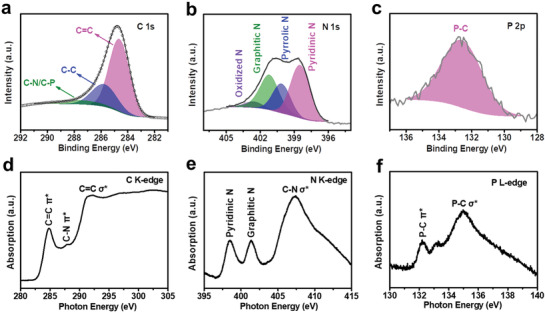
Spectroscopic characterizations of N,P‐mC. a–c) XPS spectra of N,P‐mC; d–f) XANES spectra of N,P‐mC.

Next, the electrocatalytic activity of N,P‐mC and N‐mC for CO_2_RR were investigated and compared. Experiments were performed in a home‐built H‐type electrochemical cell filled with 0.5 m NaHCO_3_ (see the Supporting Information for details). Catalyst powders were mixed with Ketjenblack carbon and Nafion binder, and loaded onto carbon fiber paper as the current collector. When the electrolyte is saturated with Ar, both N,P‐mC and N‐mC exhibit cathodic current density due to HER (**Figure** **3**a). Their onset potential (defined as the potential to reach *j* = 0.5 mA cm^−2^) is measured to be −0.46 V (vs RHE, the same applies hereinafter) and −0.63 V, respectively. When the electrolyte is saturated with CO_2_, HER becomes suppressed (as we would discuss later), and larger cathodic current density is recorded as a result of CO_2_RR. The onset potential of N,P‐mC and N‐mC is improved to −0.34 and −0.48 V, respectively. At −0.6 V, the cathodic current density of the former (9.9 mA cm^−2^) is over five times larger than that of the latter (1.4 mA cm^−2^). This immediately highlights the great advantage of N,P‐codoping over N‐doping alone since N,P‐mC and N‐mC have very similar microstructures.

**Figure 3:**
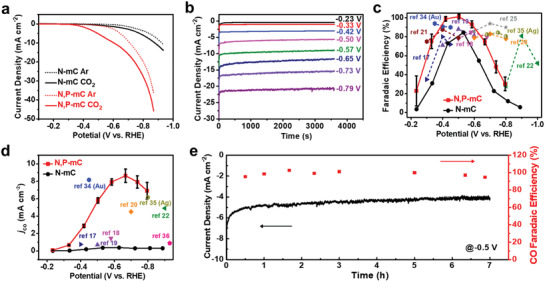
Electrochemical performance of N,P‐mC for CO_2_RR. a) Polarization curves of N,P‐mC and N‐mC in Ar‐ or CO_2_‐saturated 0.5 m NaHCO_3_; b) chronoamperometric responses of N,P‐mC at different potentials at indicated; c) CO faradaic efficiency of N,P‐mC and N‐mC in comparison with the peak faradaic efficiency of earlier reports; d) CO partial current density of N,P‐mC and N‐mC in comparison with *J*_CO_ of earlier reports at the potential corresponding to their peak selectivity; e) long‐term chronoamperometric stability and corresponding CO faradaic efficiency of N,P‐mC at −0.5 V.

To determine the CO_2_RR product and selectivity, we carried out chronoamperometric (*j* ∼ *t*) tests at a few selected potentials as shown in Figure 3b. Gaseous products were detected using online gas chromatography (GC) during the tests, and liquid products were analyzed using nuclear magnetic resonance (NMR) at the end of the tests. For both N,P‐mC and N‐mC, CO is found to be the predominant product from CO_2_RR together with a minor amount of H_2_ from HER, in line with many previous studies on N‐doped carbons.^[^ ^16^, ^17^, ^18^, ^19^, ^20^, ^21^ ^]^ Remarkably, the CO product of N,P‐mC is reproducibly detected at as early as −0.23 V (corresponding to a small overpotential of *η* = 120 mV) with measured selectivity of ≈20% (Figure 3c). Since the onset, its CO selectivity quickly rises and reaches close to unity between −0.4 and −0.6 V before HER finally sets in. By stark contrast, the CO faradaic efficiency of N‐mC exhibits a later onset (<−0.3 V) and a lower peak value (84% at −0.53 V), which are generally consistent with earlier reports on singly N‐doped carbons.^[^ ^17^, ^18^, ^19^ ^]^ Control experiment shows that when the electrolyte is saturated with Ar instead of CO_2_, zero CO is produced (Figure S8, Supporting Information). This unambiguously supports that CO is reduced from CO_2_, not HCO_3_^−^ or other carbon sources. Figure 3c also plots the CO faradaic efficiencies reported in recent literatures on heteroatom‐doped carbons^[^ ^17^, ^18^, ^19^, ^20^, ^21^, ^22^, ^25^ ^]^ together with the most representative works on Au and Ag (well known for producing CO).^[^ ^34^, ^35^ ^]^ Comparison among them shows that our N,P‐mC clearly stands out for its higher faradaic efficiency over a broader potential window. None of other metal‐free electrocatalysts has maximum selectivity over 90% except for N,S‐codoped carbon nanofibers, whose peak value (94%), unfortunately, is not achieved until at −0.7 V.^[^ ^25^ ^]^ This unambiguously highlights the unique effect of N,P‐codoping compared to single doping or other codoping.

Furthermore, we derived the potential‐dependent CO partial current density (denoted as *j*_CO_) of N,P‐mC and N‐mC as shown in Figure 3d. N‐mC with N‐doping alone exhibits a very low CO partial current density (<0.4 mA cm^−2^) over the entire potential window in CO_2_‐saturated 0.5 m NaHCO_3_, which is generally consistent with earlier reports on singly N‐doped carbons.^[^ ^16^, ^17^, ^18^, ^19^, ^36^ ^]^ Upon the incorporation of P dopants in N,P‐mC, the CO partial current density is dramatically improved–it takes off with 0.42 mA cm^−2^ at −0.23 V and culminates with ≈8 mA cm^−2^ at −0.65 V (*η* = 0.54 V). The ≈20‐fold increase evidences the advantage of N,P codoping over singly N doping. Also plotted in Figure 3d are the CO partial current densities of previous studies at the potential corresponding to their peak CO selectivity.^[^ ^17^, ^18^, ^19^, ^20^, ^22^, ^34^, ^35^, ^36^ ^]^ Most previous works on metal‐free electrocatalysts report much smaller CO partial current density generally in the range of 0–2 mA cm^−2^. Even though our material may be still some distance away from the states of the art, it represents the best metal‐free CO_2_RR material as far as we are aware. In addition, we explore the effect of different P doping levels and find that the CO partial current density generally increases with the P at% (Figure S9, Supporting Information).

Besides its great activity and selectivity, N,P‐mC has very decent stability. Figure 3e depicts its chronoamperometric response when biased at −0.5 V (corresponding to its peak CO selectivity) for 7 h. The total cathodic current density experiences an initial decay, gradually levels off, and still maintains 4.2 mA cm^−2^ at the end of the stability test. Its corresponding CO selectivity retains over ≈95% throughout the test. SEM characterization of N,P‐mC after the stability test shows that the original concave dodecahedral shape is well preserved (Figure S10, Supporting Information). No noticeable change in the N and P doping nature or level is observed from XPS analysis (Figure S11, Supporting Information).

In order to better understand the synergy between N and P during CO_2_RR, we carried out density functional theory (DFT) calculations using the computational hydrogen electrode (CHE) model (see the Supporting Information for details). By analyzing the thermodynamics of different proton‐coupled‐electron‐transfer (PCET) steps and evaluating the theoretical overpotential, DFT computation is capable of predicting and elucidating the catalytic behavior of electrocatalyst materials at the most fundamental level.^[^ ^37^, ^38^, ^39^ ^]^ In our work, several possible configurations were initially considered and screened for both single doping and codoping. Their total energies were calculated and compared. Six most stable geometry structures with the lowest energy are selected and depicted in **Figure** **4**a, denoted as graphitic‐N, pyridinic‐N, P, graphitic‐N+metaP, graphitic‐N+paraP, pyridinic‐N+metaP. Electrochemical CO_2_ reduction to CO through adsorbed *COOH and *CO intermediates was then simulated on these model configurations. Their computed energy profiles are summarized in Figure 4b. Previous studies indicate that pyridinic N is the most favorable catalytic site in singly N‐doped carbons for CO_2_RR.^[^ ^16^, ^17^, ^18^ ^]^ This is also what we find out here from our DFT calculations. The first proton‐coupled electron transfer of CO_2_RR to form *COOH is highly endothermic (+1.12 eV) on graphitic‐N, whereas the free energy for the *COOH formation on pyridinic‐N is considerably lowered to +0.33 eV. This very first step is the rate determining step for both of them. Of note, P‐doping is expected to have a different effort due to the additional orbital and higher electron‐donating ability of P. The hybridization between P 3p and C 2p orbitals results in a pyramidal‐like structure with more exposed P atoms for binding CO_2_ and reaction intermediates.^[^ ^40^ ^]^ Indeed, the *COOH adsorption free energy is calculated to be +0.25 eV on singly P‐doped graphite. Unfortunately, the further reduction of *COOH to *CO is found to be more endothermic (+0.38 eV) and thereby is the rate determining step. P‐doping alone is no obviously advantageous than N‐doping alone.

**Figure 4:**
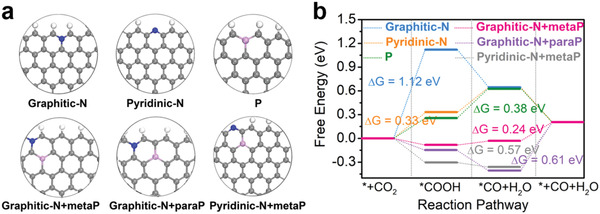
Synergy of N and P codoping revealed by DFT simulations. a) Optimized geometry structures with different heteroatom doping considered in our calculations; b) energy profiles of CO_2_RR to CO on the six different model configurations.

Very interestingly, we find that the synergistic coupling between N and P could significantly enhance the adsorption of *COOH and *CO with P being the preferred binding site. The adsorption free energy of *COOH is calculated to be −0.30, −0.15, and −0.08 eV on pyridinic‐N+metaP, graphitic‐N+paraP and graphitic‐N+metaP respectively, while that of *CO is calculated to be −0.36, −0.41, and −0.03 eV on pyridinic‐N+metaP, graphitic‐N+paraP and graphitic‐N+metaP respectively. For all of them, the first step toward *COOH formation is spontaneous. Due to the relatively strong binding strength of *CO, its desorption from catalyst surface then becomes the rate determining step. The most active configuration is found to be graphitic‐N+metaP. It is predicted to have a small overpotential of 0.24 V, which is superior to singly N‐doping and reasonably close to our experimentally measured onset overpotential. The optimized adsorption configurations of *COOH and *CO on graphitic‐N+metaP are illustrated in Figure S12 (Supporting Information). Even though there still lacks solid experimental support of the existence of graphitic‐N+metaP, we believe that its formation is statistically possible because the product is derived from PA‐ZIF‐8 with direct phosphate‐N interactions so that P is very likely located close to N in our N,P‐mC. In addition, our computations evidence that all the six model configurations adsorb *H either too strongly (∆G_H_<−0.5 eV) or too weakly (∆*G*_H_ > 0.5 eV) (Figure S13, Supporting Information). This indicates that CO_2_RR is thermodynamically favored over HER on N‐doped, P‐doped or N,P‐codoped carbons. The latter reaction would become suppressed in the presence of CO_2_, consistent with our experimental observation.

In summary, we demonstrated that N,P‐codoping could dramatically improve the electrocatalytic performance of carbonaceous materials for CO_2_RR. N,P‐codoped mesoporous carbon was prepared from the phytic acid functionalization and subsequently high‐temperature NH_3_ annealing of ZIF‐8. The resultant product consisted of concave dodecahedra with a large surface area of 2325 m^2^ g^−1^ and a large mesopore volume of 1.25 cm^3^ g^−1^. The successful incorporation of both N and P dopants was verified by XPS and XANES characterizations. In CO_2_‐saturated 0.5 m NaHCO_3_, N,P‐mC enabled the selective CO_2_ reduction to CO with a small overpotential, excellent selectivity (close to unity between −0.4 and −0.6 V), large CO partial current density (≈8 mA cm^−2^) and decent stability. Most impressively, its selectivity and CO partial current density are far superior to existing singly doped or codoped carbons free of metal. The great performance was then rationalized via DFT calculations showing that the synergy between N and P significantly enhanced the adsorption of both *COOH and *CO intermediates.

## Conflict of Interest

The authors declare no conflict of interest.

## Supporting information

Supporting Information
