# Supplementary material for: Toward Highly Selective Electrochemical CO2 Reduction using Metal‐Free Heteroatom‐Doped Carbon
Source: Adv Sci (Weinh). 2020 Jun 30;7(16):2001002. doi: 10.1002/advs.202001002 (PMC7435239; doi:10.1002/advs.202001002)
Supplement: Supplementary file 1 — Supporting Information [file ADVS-7-2001002-s001.pdf]

WILEY-VCH

Copyright WILEY-VCH Verlag GmbH & Co. KGaA, 69469 Weinheim, Germany, 2020.

## Supporting Information

# Towards Highly Selective Electrochemical $CO_2$ Reduction using Metal-free Heteroatom-doped Carbon

*Binbin Pan*†*, Xiaorong Zhu*†*, Yunling Wu, Tongchao Liu, Xuanxuan Bi, Kun Feng, Na Han, Jun Zhong, Jun Lu,* Yafei Li* and Yanguang Li**

Mr. B. B. Pan, Dr. Y. L. Wu, Mr. K. Feng, Dr. N. Han, Prof. J. Zhong, and Prof. Y. G. Li
Institute of Functional Nano & Soft Materials (FUNSOM)
Jiangsu Key Laboratory for Carbon-Based Functional Materials and Devices
Soochow University, Suzhou 215123, China; E-mail: yanguang@suda.edu.cn

Dr. T. Liu, Dr. X. Bi and Dr. J. Lu
Chemical Sciences and Engineering Division, Argonne National Laboratory, Lemont, IL 60439, USA; E-mail: junlu@anl.gov

Miss. X. R. Zhu, Prof. Y. F. Li
College of Chemistry and Materials Science, Nanjing Normal University, Nanjing 210023, China; E-mail: liyafei@njnu.edu.cn

Dr. T. Liu
School of Advanced Materials, Peking University, Shenzhen Graduate School, Shenzhen 518055, China

† These two authors contribute equally.

**Experimental Section**

*Preparation of N-mC and N,P-mC:* In a typical synthesis, ZIF-8 was first prepared by mixing 100 mL methanol solution of 50 mM zinc nitrate hexahydrate ($Zn(NO_3)_2$•$6H_2O$, from Aldrich, 98%), 3.2 mL of 1-methylimidazole ($C_4H_6N_2$, from Aldrich, ⩾99%) and 100 mL methanol solution of 200 mM 2-methylimidazole ($C_4H_6N_2$, from Aldrich, ⩾99%). The solution stood for 12 h at 25 °C. Resultant white precipitates were collected by centrifugation, washed with anhydrous methanol and dried in a vacuum oven at 55 °C. To prepare PA-ZIF-8, 120 mg of ZIF-8 powder was dispersed in 50 mL of methanol containing different amounts (~20 to ~190 mg, see Table S1) of phytic acid ($C_6H_{18}O_{24}P_6$, ⩾70% in $H_2O$, from Aladdin, shorted as PA) and was mechanically agitated for 8 h at RT. The solid powder was then recollected by configuration and dried in a vacuum oven at 55 °C. The final product was prepared by annealing PA-ZIF-8 at 1050 °C under 10% $NH_3$/Ar for 1 h. Note that product prepared with ~50 mg of phytic acid was refered as the standard sample and described as N,P-mC in our manuscript. N-mC was prepared by directly annealing ZIF-8 at 1050 °C under 10% $NH_3$/Ar for 1 h.

*Material characterizations:* SEM was performed on a Supera 55 Zeiss scanning electron microscope. TEM and STEM were performed on an FEI Tecnai F20 transmission electron microscope operating at an acceleration voltage of 200 kV. XRD patterns were collected on a PANalytical X-ray diffractometer using Cu Kα radiation (λ = 0.154 nm). Raman spectra were measured using a LabRAM-HR Raman spectrometer with a 633 nm laser excitation wavelength. XPS analysis was conducted on an ULTRA DLD XPS spectrometer. C K-edge, N K-edge and P $L_{2,3}$-edge soft XANES measurements were carried out at the beamline 20 A of Taiwan Light Source. The energy scales of C K-edge and N K-edge XANES were calibrated against SiC and BN standards, respectively. Surface areas were measured using a Micromeritics ASAP 2020 surface area analyzer.

*$CO_2RR$ measurements:* Electrochemical experiments were carried out in an airtight two-compartment H-cell. To prepare the working electrode, 1 mg of N,P-mC (or N-mC) and 0.5 mg of Ketjenblack carbon were dispersed in 200 μL of ethanol, 50 μL of ultrapure water and 6 μL of 5 wt% Nafion solution, and subjected to vigorous ultrasonication for over half an hour till the formation of a homogeneous catalyst ink. The ink was then carefully dropcast onto a 1 × 1 $cm^2$ carbon fiber paper (HCP020P, from HESEN) to reach a catalyst loading of ~1 $mg/cm^2$. $CO_2$RR measurements were performed in the standard three electrode configuration controlled by a CHI660c potentiostat. the catalyst-loaded working electrode and a saturated calomel reference electrode were placed in the cathodic compartment, while a graphite counter electrode was placed in the anodic compartment. The two compartments were separated by a Nafion membrane in the middle. The electrolyte was 0.5 M $NaHCO_3$ solution pre-saturated with either Ar (pH = 8.4) or $CO_2$ (pH = 7.2). During electrochemical measurements, a flow of 20 sccm of $CO_2$ was continuously bubbled into the electrolyte to maintain its saturation. Potential readings were converted to the RHE scale and corrected for the ohmic loss. Gaseous reduction products were analyzed using an on-line gas chromatograph (GC, Aligent 7890B) equipped with a molecular sieve 5A and two porapak Q columns. The concentration of CO was analyzed by a flame ionization detector (FID) and the concentration of $H_2$ was analyzed by a thermal conductivity detector (TCD). Their Faradaic efficiency was calculated as follows:

$$FE(\%) = \frac{Q_{co}}{Q_{tot}} \times 100\% = \frac{\left(\frac{v}{60\ s/min}\right) \times \left(\frac{y}{24000\ cm^3/mol}\right) \times N \times F \times 100\%}{j}$$

where $F$ is the Faraday constant (96500 $C{\cdot}mol^{-1}$), $v$ is the $CO_2$ flow rate (20 sccm), $y$ is the measured concentration of product in 1 mL sample loop based on calibration of the GC with a standard gas, $N$ (=2) is the number of electrons transferred in the reaction process, $j$ is the corresponding current at different potentials.

*Computational methods:* All theoretical calculations were performed using Vienna ab initio simulation packages (VASP).[1] The exchange-correlation interaction was described by the generalized gradient approximation (GGA) with the Perdew–Burke–Ernzerhof (PBE) functional.[2] The plane wave cutoff was set to 500 eV, with the convergence of energy and force set to $1\times10^{-5}$ and 0.01 eV·Å$^{-1}$, respectively. To avoid the interaction between two adjacent layers, the vacuum thickness was set to 15 Å. The model of N,P co-doped system was set in a supercell of a graphene ribbon with zigzag C atoms edges. The edge C atoms were saturated with H atoms.The Brillouin zone was sampled by a 5×1×1 k-point grid with the Monkhorst–Pack scheme for all structural optimization. The CHE model[3] was used to take into account the effect of the applied potential on free energy changes, where the chemical potential of the proton-electron pair was set to be equivalent to that of gas-phase $H_2$ in the standard condition.

## References

[1] G. Kresse, J. Furthmüller, *Phys. Rev. B* **1996**, *54*, 11169.
[2] J. P. Perdew, K. Burke, M. Ernzerhof, *Phy. Rev. Lett.* **1996**, *77*, 3865.
[3] A. A. Peterson, F. Abild-Pedersen, F. Studt, J. Rossmeisl, J. K. Nørskov, *Energy Environ. Sci.* **2010**, *3*, 1311.

**Table S1.** Preparation conditions of N,P-mC-1/2/3 and N-mC.

| **Electrocatalysts** | **Added amount of phytic acid in the second step** |
|---|---|
| N-mC | No phytic acid |
| N,P-mC-1 | ~20 mg of phytic acid |
| N,P-mC-2 (the standard sample) [a] | ~50 mg of phytic acid |
| N,P-mC-3 | ~190 mg of phytic acid |

[a] Please note in our manuscript, N,P-mC and N,P-mC-2 are used interchangeably.

**Table S2.** The N at% and P at% of N,P-mC-1/2/3 and N-mC from XPS.

| **Electrocatalysts** | **N at%** | **P at%** |
|---|---|---|

| | | |
|---|---|---|
| N-mC | 4.2 | - |
| N,P-mC-1 | 5.3 | 0.04 |
| N,P-mC-2 (or N,P-mC) | 5.3 | 0.11 |
| N,P-mC-3 | 4.1 | 0.24 |

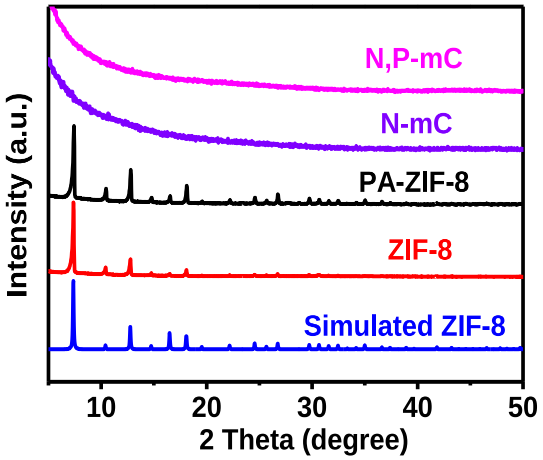


**Figure S1.** XRD patterns of simulated ZIF-8, ZIF-8, PA-ZIF-8, N-mC and N,P-mC.

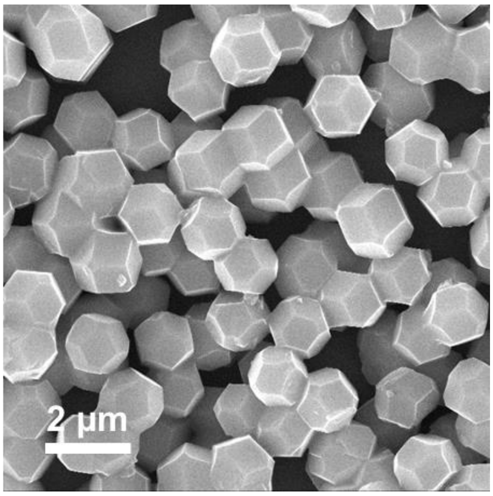


**Figure S2.** SEM image of PA-ZIF-8.

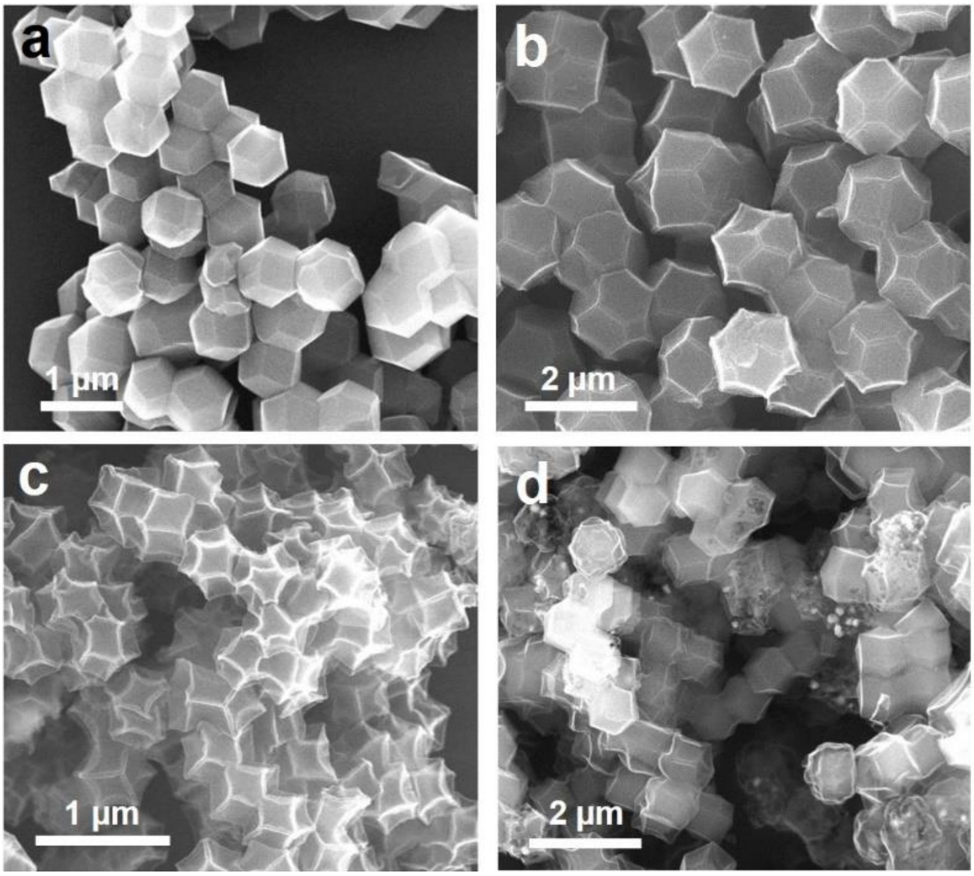


**Figure S3.** SEM images of (a) N-mC, (a) N,P-mC-1, (b) N,P-mC-2 and (c) N,P-mC-3.

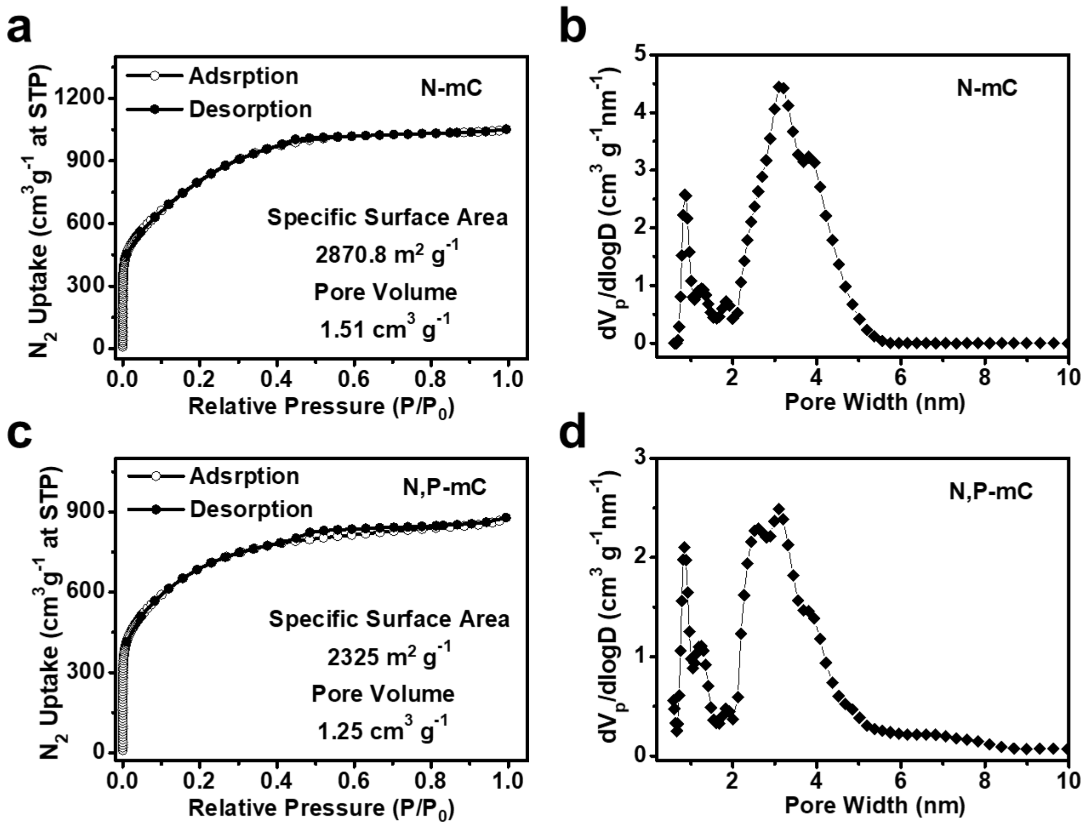


**Figure S4.** (a,c) $N_2$ adsorption-desorption isotherms of N-mC and N,P-mC, and (b,d) their corresponding pore size distribution curves calculated using the NLDFT method.

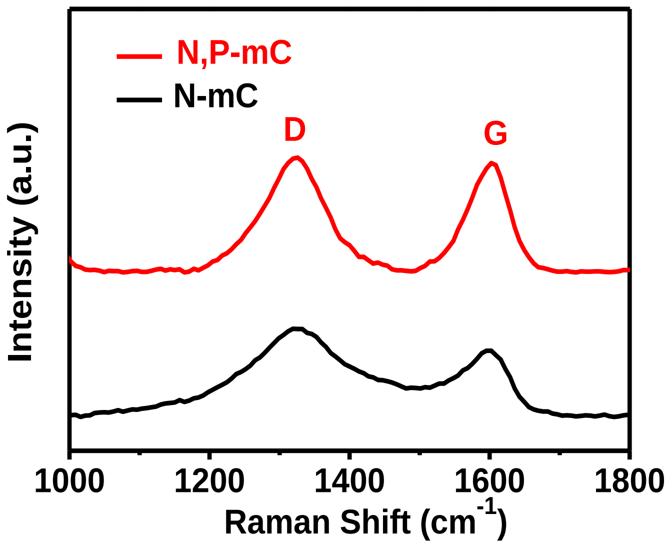


**Figure S5.** Raman spectra of N-mC and N,P-mC.

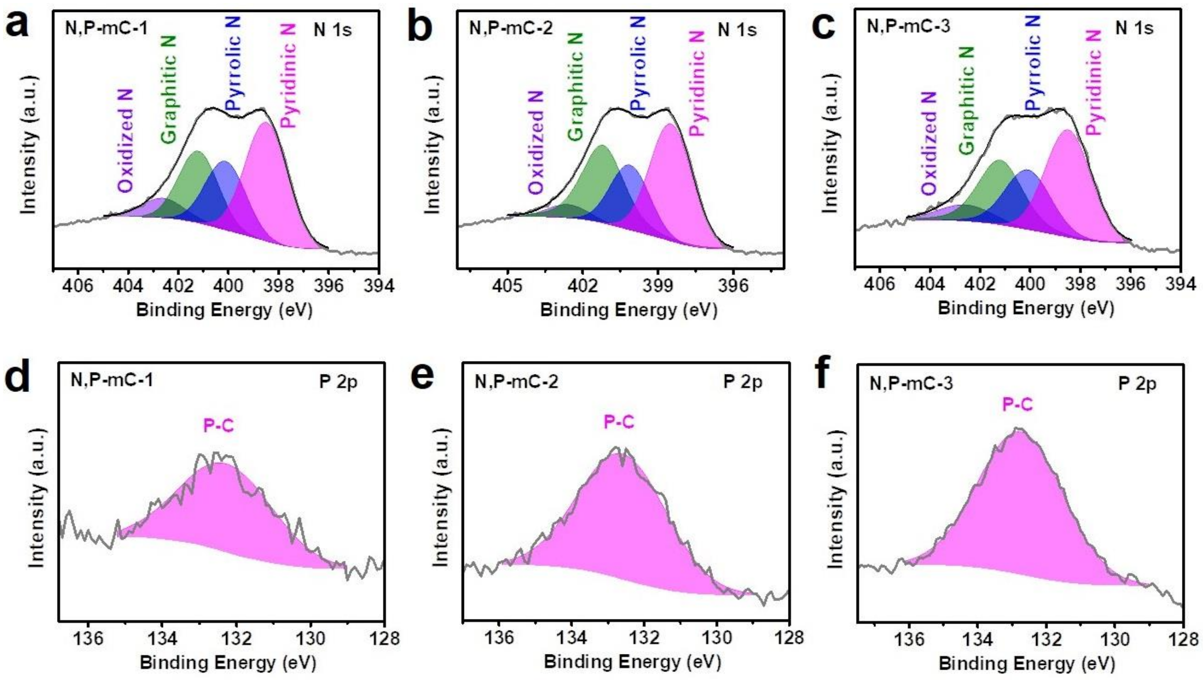


**Figure S6.** (a-c) N 1s XPS spectra and (d-f) P 2p XPS spectra of N,P-mC-1/2/3.

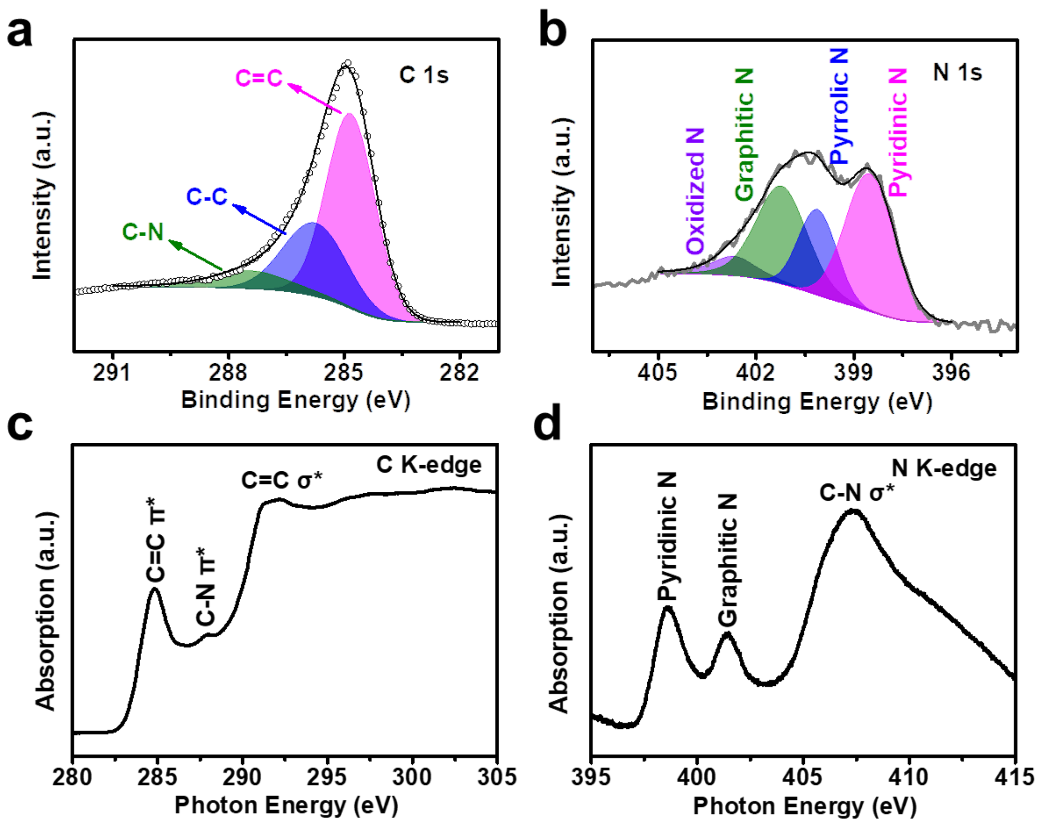


**Figure S7.** (a,b) XPS spectra and (c,d) XANES spectra of N-mC.

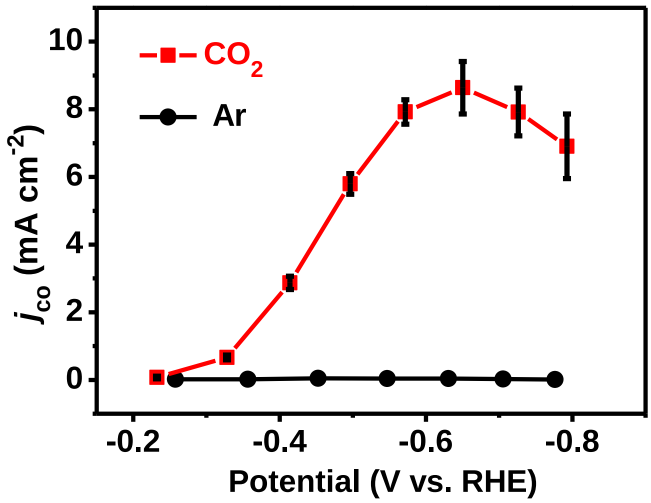


**Figure S8.** CO partial current density of N,P-mC in $CO_2$-saturated or Ar-saturated 0.5 M $NaHCO_3$.

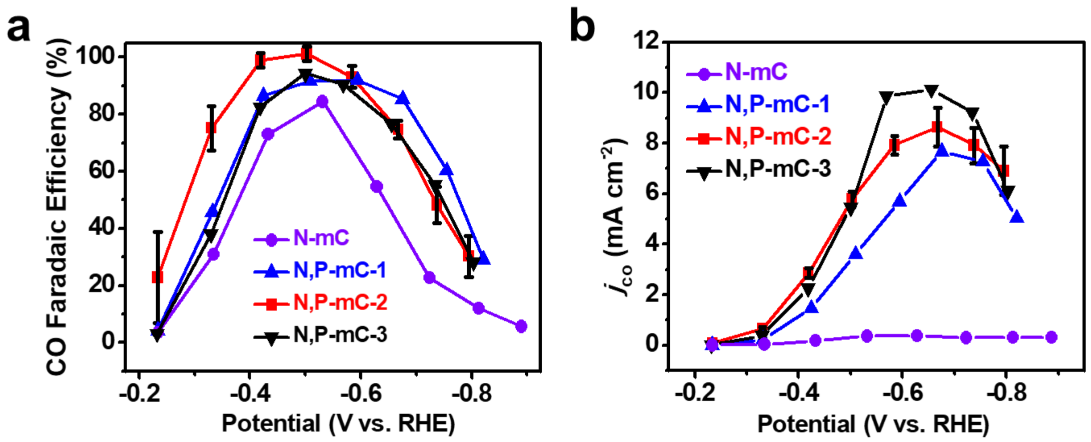


**Figure S9.** (a) CO Faradaic efficiency and (b) CO partial current density of N,P-mC-1/2/3 and N-mC.

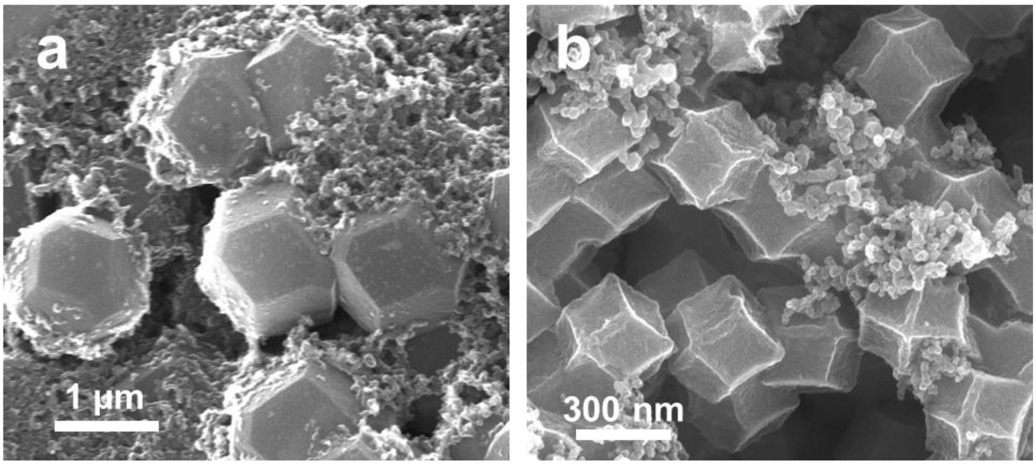


**Figure S10.** SEM images of (a) N-mC and (b) N,P-mC after the long-term chronoamperometric stability test.

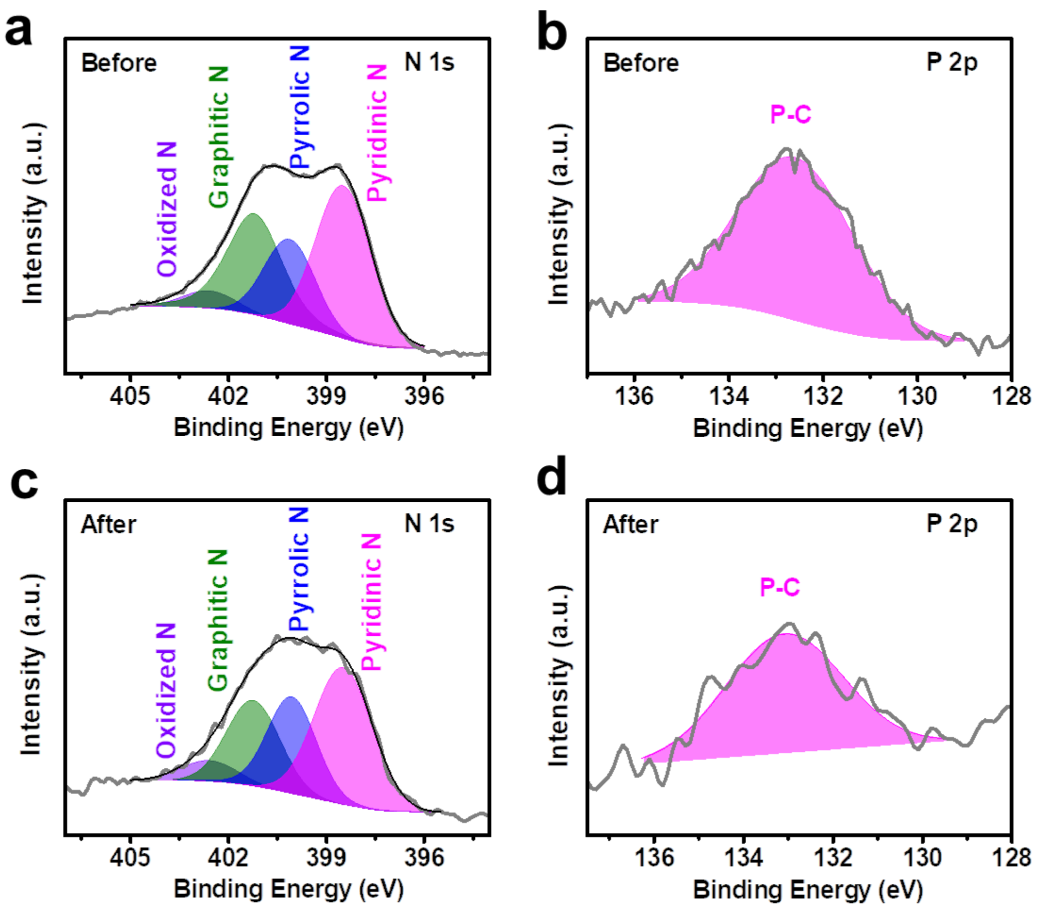


**Figure S11.** (a,b) N 1s and (c,d) P 2p XPS spectra of N,P-mC before and after the stability test.

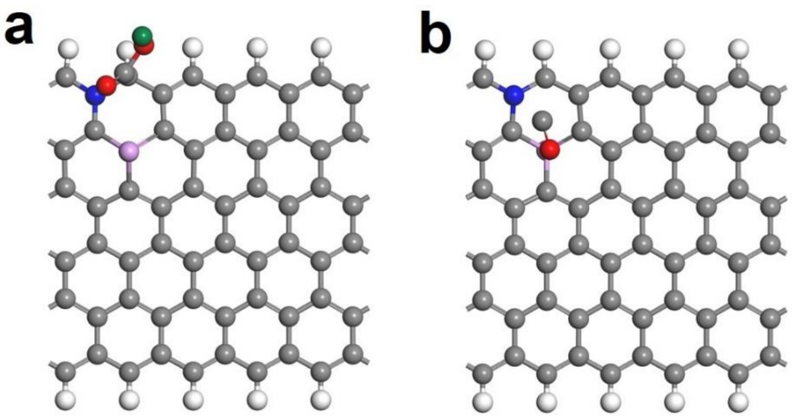


**Figure S12**. Optimized adsorption configurations of (a) *COOH and (b) *CO on graphitic-N+metaP.

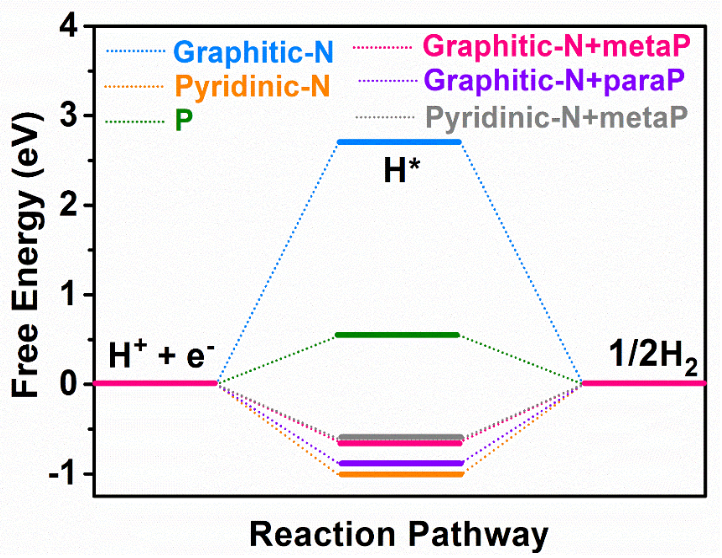


**Figure S13.** Energy profiles of HER on the six model configurations.
